# Matrix Metalloproteinases on Skin Photoaging

**DOI:** 10.1111/jocd.16558

**Published:** 2024-09-04

**Authors:** Chao Feng, Xianglong Chen, Xiuqing Yin, Yanfei Jiang, Chunyue Zhao

**Affiliations:** ^1^ Beijing Qingyan Boshi Health Management Co., Ltd. Beijing China

**Keywords:** extracellular matrix breakdown, matrix metalloproteinase (MMP), MMP inhibitors, skin aging

## Abstract

**Background:**

Skin aging is characterized by an imbalance between the generation and degradation of extracellular matrix molecules (ECM). Matrix metalloproteinases (MMPs) are the primary enzymes responsible for ECM breakdown. Intrinsic and extrinsic stimuli can induce different MMPs. However, there is limited literature especially on the summary of skin MMPs and potential inhibitors.

**Objective:**

We aim to focus on the upregulation of MMP expression or activity in skin cells following exposure to UV radiation. We also would like to offer valuable insights into potential clinical applications of MMP inhibitors for mitigating skin aging.

**Methods:**

This article presents the summary of prior research, which involved an extensive literature search across diverse academic databases including Web of Science and PubMed.

**Results:**

Our findings offer a comprehensive insight into the effects of MMPs on skin aging after UV irradiation, including their substrate preferences and distinct roles in this process. Additionally, a comprehensive list of natural plant and animal extracts, proteins, polypeptides, amino acids, as well as natural and synthetic compounds that serve as inhibitors for MMPs is compiled.

**Conclusion:**

Skin aging is a complex process influenced by environmental factors and MMPs. Research focuses on UV‐induced skin damage and the formation of Advanced Glycosylation End Products (AGEs), leading to wrinkles and impaired functionality. Inhibiting MMPs is crucial for maintaining youthful skin. Natural sources of MMP inhibitor substances, such as extracts from plants and animals, offer a safer approach to obtain inhibitors through dietary supplements. Studying isolated active ingredients can contribute to developing targeted MMP inhibitors.

## 1 Introduction

The skin, which consists of the outermost layer known as the epidermis, the thicker and more elastic middle layer called the dermis, and the inner layer of subcutaneous tissue, serves as the body's largest organ that facilitates our interaction with the environment [1]. The process of skin aging is a consequence of the combined effects of intrinsic and extrinsic factors, encompassing exposure to solar radiation, air pollution, tobacco consumption, inadequate nutritional intake, and inappropriate use of cosmetics [2]. Light plays a pivotal role in sustaining life and exhibits a close association with skin health. In 1969, the initial hypothesis proposed that sun exposure could potentially result in skin damage and accelerate aging [3]. Sun exposure encompasses ultraviolet (UV) radiation, which can elicit cutaneous heat stress.

The extracellular matrix (ECM), which fills intercellular spaces and acts as a communication conduit between cells and organisms, encompasses a multitude of molecules within the skin, including proteoglycans, glycosaminoglycans, structural proteins (such as collagen and elastin), adhesion proteins (such as fibronectin and laminin), along with proteases such as matrix metalloproteases (MMPs) [4]. Imbalance in ECM components is commonly associated with skin aging. The restoration of ECM levels to their normal state has been widely acknowledged as an effective approach for mitigating the process of skin aging. Strategies encompass the inhibition of MMP function, along with the stimulation of collagen and elastin secretion.

MMPs play a pivotal role in the regulation of ECM, and elevated expressions and/or activities of MMPs are frequently associated with skin aging. This review comprehensively examines the roles of MMPs in skin aging, provides an extensive list of substances capable of inhibiting MMP activity to decelerate skin aging, reviews specific targeted MMPs along with their potential signaling pathways, and discusses the prospects of these substances as food, medicine, or skincare products for mitigating skin aging.

## 2 Collagen and Elastin are the Most Important Components of Skin ECM

Collagen and elastin are the primary constituents of the ECM that provide structural support to the skin. Collagen accounts for 30% of the total ECM proteins in our bodies, imparting strength and stability to various tissues including skin, muscles, bones, and connective tissues. Type I and type III collagens are predominantly localized in the skin, with type I representing approximately 90% of body collagen content. The dermis, comprising around 70%–80% collagen composition, confers remarkable structural integrity to the skin. Moreover, collagen fibrils possess unique properties such as sliding and realignment capabilities that ensure maintenance of skin integrity while allowing deformation without causing damage [5]. Elastin, despite being a minor constituent, plays a crucial role in endowing the skin with elasticity by facilitating its capacity to revert to its original state following external forces [5].

Skin aging is intricately associated with the dysregulation between the accumulation and degradation of ECM components, which provide structural and functional support to the skin tissue. In this review, we consider ECM degradation as an indicator of collagen and elastin fiber breakdown. In youthful, healthy skin, collagen synthesis occurs continuously while damaged or excessive collagen undergoes degradation. However, during the process of aging, there is a decline in collagen synthesis capacity leading to gradual loss of collagen content. This highlights the significance of maintaining a balanced turnover of ECM in delaying skin aging.

## 3 The Proteases that Degrade the ECM

The degradation of the ECM is primarily mediated by three types of proteases, namely MMPs, plasmin, and cathepsins. Among these proteases, MMPs exert significant influence on ECM degradation.

MMPs are zinc‐dependent enzymes initially discovered in the tails of tadpoles [6]. MMPs play a pivotal role in maintaining the homeostasis of the ECM in the skin by effectively degrading collagen and potentially facilitating elastin degradation. Furthermore, they actively participate in diverse biological and physiological processes including embryogenesis, morphogenesis, angiogenesis, and wound healing [4]. Dysregulated MMP activity is implicated in a myriad of pathological conditions, encompassing inflammation, tissue damage, fibrosis, aberrant angiogenesis, matrix destabilization, microglial activation, autoimmune diseases, and carcinogenesis [7].

MMPs are synthesized by a variety of skin cell types, including fibroblasts, keratinocytes, macrophages, endothelial cells, mast cells, and eosinophils [8]. In humans, there are 25 distinct MMPs that can be categorized based on their substrate specificity or cellular localization: collagenases (MMP‐1, MMP‐8, MMP‐13, and MMP‐18), gelatinases (MMP‐2 and MMP‐9), stromelysins (MMP‐3 and MMP‐10), matrilysins (MMP‐7 and MMP‐26), furin‐containing secreted MMPs (MMP‐11, MMP‐21, and MMP‐28), membrane‐type I MT‐MMPs (MMP‐14, MMP‐15, MMP‐16, and MMP‐24), GPI anchored MT‐MMP (MMP‐17 and MMP‐25), and type II MT‐MMP like the case of MMP‐23 [9], as well as other miscellaneous forms of MMP (MMP‐12, MMP‐19, MMP‐20, MMP‐22, and MMP‐27).

The proteolytic enzyme plasmin plays a crucial role in the dissolution of blood clots by specifically degrading fibrin within the body. Moreover, plasmin has been reported to exhibit its capability of degrading various basement membrane proteins, such as laminin and fibronectin, both of which are components of the skin ECM [10, 11]. The activation of MMPs, including MMP‐1, MMP‐2, MMP‐3, MMP‐9, MMP‐13, and MMP‐14, is also facilitated by plasmin‐mediated degradation of the ECM [12]. Additionally, specific members within the cathepsin family have been identified as degraders of ECM proteins, specifically targeting type I collagen, type IV collagen, fibronectin, and laminin [13, 14]. In this review, our primary focus will be on discussing MMPs, with no further discussion of plasmin and cathepsins.

## 4 Exposure to UV Radiation Induces the Expression of MMPs

Ultraviolet exposure, extensively researched, is an integral facet of daily life and demands our attention. The ultraviolet radiation reaching the earth's surface encompasses both short and long wavelengths; UVA (320–400 nm) and UVB (280–320 nm) are the two most extensively investigated types. UVA represents the predominant UV ray that reaches the Earth's surface, while UVB exhibits higher intensity. Exposure to UV radiation can lead to sunburn, DNA damage, and suppression of the immune system. Prolonged exposure to UV induces molecular and cellular damage to normal skin structure, resulting in premature skin aging (photoaging), as well as the development of skin cancers such as melanoma, basal cell carcinoma (BCC), and squamous cell carcinoma (SCC) [15, 16].

UV radiation is widely acknowledged as the primary determinant of skin photoaging, characterized by epidermal thickening, textural coarseness, pronounced wrinkles, and dyspigmentation patterns [3]. The involvement of UV radiation in the upregulation of MMPs in fibroblasts has been hypothesized, with both UVA and UVB radiations capable of inducing the expression and secretion of MMPs [17, 18], and enhanced MMP activity is considered a pivotal factor contributing to age‐related skin aging. Moreover, UV radiation induces an excessive generation of reactive oxygen species (ROS), which bind to cytokine receptors on the cell membrane and activate mitogen‐activated protein kinase (MAPK) family proteins, including extracellular signal‐regulated kinases (ERK). ERK stimulates the expression of c‐Fos, which forms AP‐1 with c‐Jun and binds to MMP promoters for transcriptional regulation [19, 20, 21]. Additionally, UVB irradiation can induce the upregulation of a membrane‐bound metalloprotease known as neutral endopeptidase (NEP), or neprilysin, which is involved in the degradation and disruption of elastin's three‐dimensional structure, ultimately resulting in skin wrinkling or sagging [22, 23].

Studies have demonstrated that MMPs exhibit distinct responses to different wavelengths of UV radiation, particularly in relation to specific types of skin cells. Specifically, it has been found that UVB rays (280–320 nm) more readily induce MMP‐1, MMP‐3, and MMP‐10 compared to UVA1 rays (long UVA 340–400 nm). Both UVB and UVA1 were observed to similarly induce MMP‐9; however, it is noteworthy that UVA1 exhibited potent induction of MMP‐12 [24]. Interestingly, exposure of human epidermal keratinocytes (NHEK) cells to UVA radiation at a dose of 30 J/cm^2^ resulted in the significant inhibition of both MMP‐2 and MMP‐9 activities [15], which contrasts with the response observed in fibroblasts.

**FIGURE 1:**
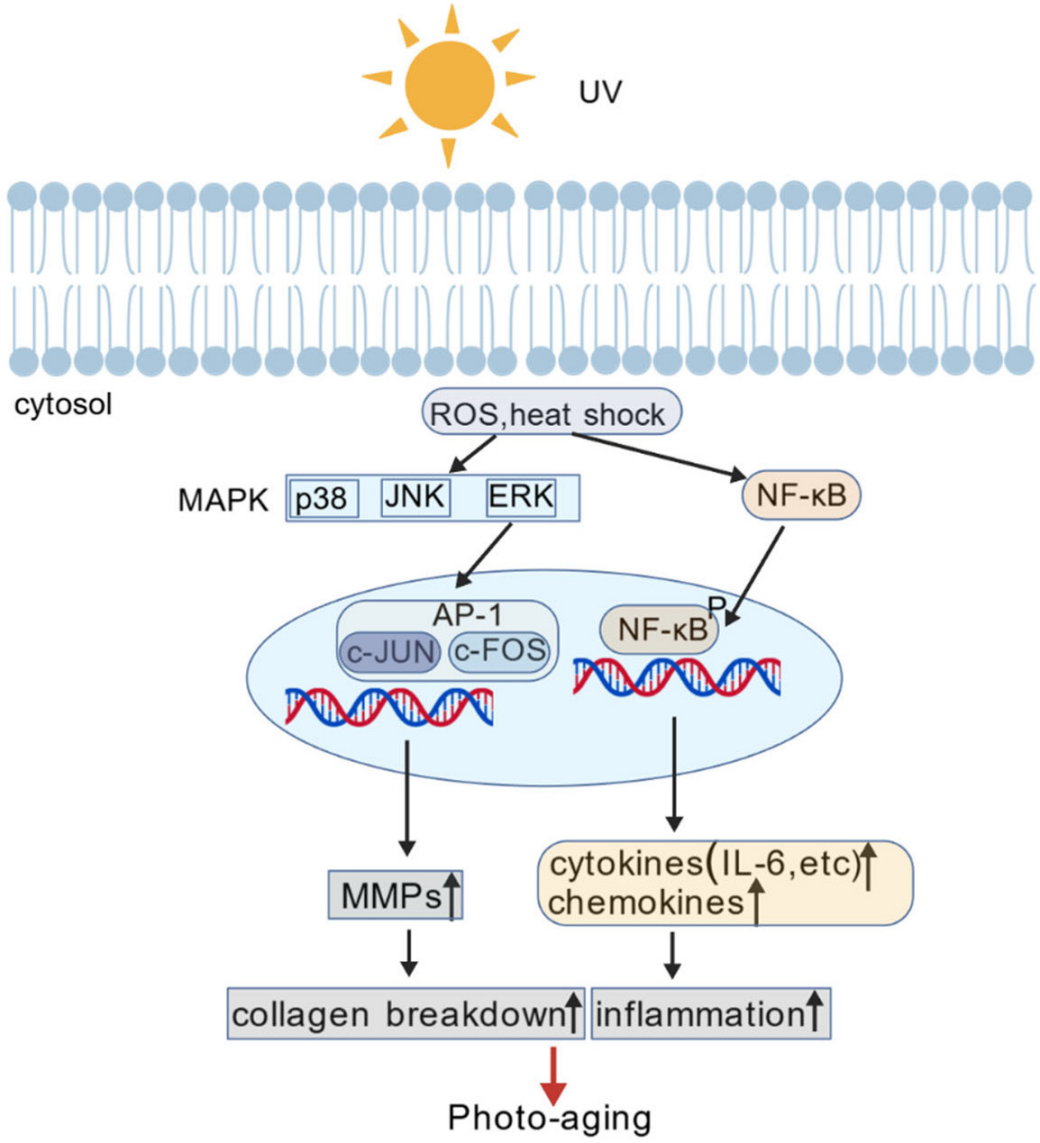
UV‐induced MMP activation and photoaging.

Sun exposure not only results in the presence of UV radiation but also induces an elevation in skin temperature, thereby causing thermal stimulation. Previous studies have demonstrated that heat shock triggers the activation of MMP‐1 and MMP‐3 (but not MMP‐2) in cultured dermal human fibroblasts through ERK and JNK signaling pathways, along with autocrine IL‐6 expression [25]. Additionally, it has been observed that thermal stimulation induces the generation of ROS in epidermal cells, thereby promoting the upregulation of tropoelastin expression while concurrently increasing fibrillin‐1 expression; however, a reduction in fibrillin‐1 expression is noted specifically in dermal cells [26]. Downstream signaling upon UV radiations and the events upon MMP activations are briefly summarized in Figure 1.

## 5 The Role of MMPs in Skin Aging

MMPs exhibit distinct preferences for various types of collagens. MMP‐1, MMP‐8, and MMP‐13 possess the capability to degrade fibrillar collagen. In contrast, MMP‐2 and MMP‐9 exhibit the ability to digest collagen types I and IV. However, it is noteworthy that MMP‐3, MMP‐10, and MMP‐11 lack the capacity to cleave collagen type I; instead, they may function as activators for other forms of matrix metalloproteinases (MMPs). Moreover, both MMP‐7 and MMP‐26 are capable of degrading collagen type IV with the former also demonstrating elastin breakdown capabilities. Lastly, MT‐MMPs such as MMP‐14 and MMP‐16 specifically target fibrillar collagen type I as their substrate [27].

Typically, MMPs exhibit distinct tissue‐specific expression patterns and substrate specificities; however, compensatory mechanisms can be observed among certain MMP isoforms [28].

### 5.1 The Collagenases

The collagenases comprise MMP‐1, MMP‐8, MMP‐13, and MMP‐18. Notably, the pivotal role of MMP‐1 in photoaging is attributed to its ability to induce UV radiation‐mediated expression and subsequent cleavage of type I and type III collagen. Moreover, stromelysins such as MMP‐3 and MMP‐9 further degrade the cleaved collagen [29]. Following UV irradiation, there was a threefold increase in the degradation of collagen within 24 h in the skin. Moreover, IL‐6 secretion from UVA‐exposed keratinocytes could enhance UVA‐induced upregulation of MMP‐1 activity in dermal fibroblasts [30].

Although MMP‐1, MMP‐8, and MMP‐13 exhibit the ability to degrade native fibrillar type I and type III collagen, recent studies have revealed that in UV‐mediated skin collagen damage, there is no significant disparity between the roles of MMP‐8 and MMP‐1. While UV light induces a slight increase in the expression of MMP‐8, another collagenase known as MMP‐13 does not demonstrate substantial upregulation in UV‐exposed skin compared to control [31]. The basal expression of MMP‐13 is typically low or undetectable in normal adult tissues; however, its expression is upregulated in response to tissue repair. TGF‐β can induce the expression of MMP‐13 while concurrently suppressing the expressions of both MMP‐1 and MMP‐3 [32]. Studies have demonstrated that while MMP‐1 and MMP‐8 primarily degrade type I and III collagens, respectively, MMP‐13 exhibits a predominant affinity for type II collagen, displaying an enhanced degradation efficiency 5–10 times greater than that of MMP‐1 [33]. Collectively, these findings suggest that MMP‐1 may serve as the primary enzyme responsible for collagen damage in human skin during photoaging processes associated with UV exposure.

### 5.2 The Gelatinases

Two members, MMP‐2 and MMP‐9, which belong to the gelatinase group, have been implicated in human skin aging. Studies have demonstrated that exposure to pollutants increases the expression level of MMP‐2 in both young and aged animals; however, only UV‐treated aged animals showed detectable levels of MMP‐9 [34]. The transcriptional regulation of most MMPs involves a complex interplay of stimulatory and suppressive factors that modulate multiple signaling pathways. Emerging evidence suggests that MMP‐2 may exhibit constitutive expression at the protein level [35]. Additionally, co‐expression of MMP‐2 and MMP‐9 in conjunction with MMP‐3 can induce the degradation of non‐collagenous constituents within the skin, including glycoproteins and proteoglycans present in the basement membrane [36, 37]. Additionally, it has been demonstrated that MMP‐9 exhibits the ability to enzymatically cleave collagen types I and III [38]. Although there is established evidence supporting MMP‐2's ability to cleave type I collagen, the extent of its collagenolytic activity remains a subject of controversy [39].

### 5.3 The Stromelysins

The members of the stromelysin family include MMP‐3, also known as stromelysin‐1, and MMP‐10, alternatively referred to as stromelysin‐2. MMP‐3 is expressed by various cell types including keratinocytes, fibroblasts, and chondrocytes both in culture and in vivo. It is primarily secreted by fibroblasts and epithelial cells along with MMP‐10 [40, 41]. Although MMP‐3 lacks the ability to cleave native type I collagen, it demonstrates a propensity for degrading other ECM proteins such as E‐cadherin, laminins, and type IV collagen. Additionally, MMP‐3 can activate collagenases and gelatinases including MMP‐1, MMP‐8, MMP‐13, and MMP‐9 [42], thereby playing a crucial role in the generation of fully active MMPs.

The pleiotropic cytokine tumor necrosis factor (TNF)‐α plays a pivotal role as an inflammatory mediator in cutaneous tissues. In fibroblasts, TNF‐α is implicated in the degradation of dermal collagen induced by photoaging [43]. Accumulating evidence suggests that TNF‐α facilitates collagen degradation by means of MMP‐1 and MMP‐3, with the potential involvement of MMP‐3 in activating MMP‐1 [44]. Notably, MMP‐3 also cleaves the precursor of TNF‐α to generate active TNF‐α, which subsequently induces the expression of MMP‐9 [45]. The induction of apoptosis by TNF‐a is significantly attenuated in mice with MMP‐3 deficiency specifically in their skin [46, 47], leading to a dramatic decrease in collagen degradation [48]. These findings suggest that while MMP‐3 does not directly degrade type I collagen, it can indirectly modulate its degradation by influencing the activity of other MMPs. Limited investigations have been conducted on the association between MMP‐10 and skin aging; however, previous research has linked it to processes such as wound healing and cell migration [49].

### 5.4 The Matrilysins

MMP‐7 and MMP‐26 are members of the matrylisin family of proteinases. The biological functions of MMP‐7 primarily involve ECM remodeling and regulation of the immune system. Active MMP‐7 cleaves ECM and basement membrane proteins, thereby activating collagenases [50]. Moreover, MMP‐7 exhibits the ability to cleave ECM substrates and augment the activities of pro‐MMP‐2 and pro‐MMP‐9. Furthermore, upon exposure to UV radiation, MMP‐7 can effectively degrade elastin [34]. In contrast, MMP‐26, also referred to as matrilysin‐2, exhibits hydrolytic activity toward collagen type IV, fibronectin, fibrinogen, and gelatin while displaying no degradation of laminin or elastin [51]. However, it is capable of activating MMP‐9 [52].

### 5.5 The Furin‐Containing MMPs

MMP‐11, MMP‐21, and MMP‐28 belong to the furin‐containing MMP group. Specifically, MMP‐11 is referred to as stromelysin‐3 and is primarily secreted by fibroblasts under both normal and pathological conditions. Interestingly, unlike other members of the MMP family, MMP‐11 exhibits an unconventional anti‐apoptotic function. Conversely, downregulation of MMP‐28 has been observed in epidermal wounds, suggesting its potential involvement in epidermal maturation [53]. To date, no reports have been published regarding the correlation between skin aging and MMP‐21.

### 5.6 The Membrane‐Type MMPs

The membrane‐type MMPs comprise a group of enzymes, namely MMP‐14, MMP‐15, MMP‐16, MMP‐24, MMP‐17, MMP‐25, and MMP‐23. Specifically, the enzymatic activity of MMP‐14 involves the cleavage of collagen types I, II, and III as well as fibronectin, vitronectin, and laminins 111 and 332. Additionally, it also cleaves fibrin molecules along with proteoglycans [54]. However, the precise identification of the key enzyme involved in skin aging remains elusive within any biological context. Remarkably, mice with MMP‐14 deletion have exhibited a severe phenotype characterized by progressive fibrosis of soft tissues [55, 56]. The mice also exhibited premature mortality, while the surviving adult mice demonstrated increased dermal thickness and tissue rigidity [55]. Importantly, this effect was confined to the skin without exerting any impact on other organs such as the kidney, liver, and lungs [57], suggesting that MMP‐14 may play a crucial role in the maintenance of collagen I in the skin. However, additional MMP‐14‐deficient mice exhibited cardiovascular anomalies and concomitant robust accumulation of type I collagen in cardiac tissues. Furthermore, researchers observed heightened senescence‐associated beta‐galactosidase (SA‐β‐Gal) activity in fibroblasts from these MMP‐14‐deficient mice, along with elevated levels of aging markers such as Hp1γ, p16INK4α, and p21CIP1/WAF1 in various tissues, indicating the presence of a senescence process. Notably, the absence of MMP‐14 also resulted in significant inflammatory response and aberrations in nuclear envelope structure within fibroblasts lacking this protein [58]. Moreover, MMP‐14 was observed to induce the activation of both MMP‐2 and MMP‐13, leading to their collaborative degradation of the ECM [59].

Currently, the specific enzyme responsible for skin aging and its associated cascade of interactions within any biological context remains unidentified. However, compelling evidence suggests that mice lacking MMP‐14 exhibit a pronounced phenotype characterized by progressive fibrosis of soft tissues in the skin [55, 56]. Mice deficient in MMP‐14 exhibit premature mortality, while adult survivors demonstrate augmented dermal thickness and tissue rigidity [55]. The observed effect is specifically confined to the skin and does not exhibit in other organs such as the kidney, liver, and lungs [57], indicating a crucial role for MMP‐14 in maintaining collagen I levels specifically within the skin. Conversely, MMP‐14‐deficient mice exhibit cardiovascular defects and significant accumulation of type I collagen in their hearts. Moreover, fibroblasts from these mice display higher SA‐β‐Gal activity and elevated expression of aging markers including Hp1γ, p16INK4α, and p21CIP1/WAF1 across various tissues—indicating an ongoing senescence process. Additionally, MMP‐14 deficiency leads to pronounced inflammatory response alongside nuclear envelope aberrations observed in fibroblasts [58]. Furthermore, MMP‐14 has been demonstrated to induce the activation of both MMP‐2 and MMP‐13, leading to their collaborative degradation of the ECM [59].

To date, there is a lack of evidence demonstrating the UV‐dependent modulation of another membrane‐type MMP‐MMP‐15 in human fibroblasts. We investigated the expression levels of MMP‐1, MMP‐2, MMP‐3, MMP‐9, MMP‐13, and MMP‐15 in human skin fibroblasts after exposure to UVA irradiation. Our observations revealed a significant upregulation of expressions for MMP‐2, MMP‐3, and MMP‐15 upon irradiation when compared with untreated control samples. Nevertheless, the degree of upregulation observed for MMP‐l was less prominent than that seen for these three specific MMPs in our study. In contrast, UVA irradiation did not exert a substantial influence on the levels of investigated MMPs, namely, MMP‐g and MMP‐i3, which may be due to their comparatively lower baseline expression levels in fibroblasts [60].

### 5.7 The Other MMPs

The other group of MMPs includes MMP‐12, MMP‐19, MMP‐20, MMP‐22, and MMP‐27. In addition to collagen degradation in the skin, alterations in elastin levels also play a pivotal role in the process of photoaging. Despite constituting only 2%–4% of the total protein content of the skin, elastin serves as the primary component responsible for rebounding and restoring functionality. Evidence indicates that macrophage MMP‐12 is the most crucial MMP involved in elastin degradation. Given that photoaging involves both fiber destruction and repair along with new fiber synthesis, mid‐product tropoelastin is more susceptible to degradation by MMP‐12 compared to insoluble elastin. Consequently, not only does MMP‐12 contribute to elastic fiber destruction but it also disrupts the repair process by degrading expressed tropoelastin. Macrophages and fibroblasts secrete MMP‐12 during acute UV‐induced skin injury [26, 61]; meanwhile, detectable levels of this enzyme have been observed in regions displaying significant elastic damage [62]. In addition to its direct involvement in elastin degradation, MMP‐12 also functions as an activator of other pro‐MMPs including pro‐MMP‐1, MMP‐2, MMP‐3, and MMP‐9 [33, 63].

## 6 MMP Inhibitors as a Promising Therapy for Skin Aging

MMPs play a pivotal role in the degradation of ECM, thereby exerting influence on both physiological and pathological processes associated with skin aging. Consequently, the development of MMP inhibitors holds immense potential for mitigating skin aging. Therefore, it is imperative to design MMP inhibitors that specifically target mechanisms related to skin aging. The initial generation of MMP inhibitors, such as Ilomastat (GM6001), Marimastat, and CGS‐27023A, exhibited limited specificity toward MMPs. Most of these first‐generation inhibitors were hydroxamic acid‐based compounds that demonstrated poor oral bioavailability and released toxic hydroxylamine during human metabolic reactions [64]. In contrast, a plethora of plants and plant extracts exhibit diverse biological activities with fewer associated side effects compared to synthetic preparations [65]. Researchers are actively investigating safer and more natural alternatives to inhibit MMPs in order to counteract skin aging caused by environmental stressors such as ultraviolet radiation or oxidative stress. Here, we present a compilation of natural substances renowned for their ability to suppress both the expression and activity of MMPs.

### 6.1 The Plant Extract, Animal Oil, and Probiotics that Inhibit MMPs

Certain bioactive compounds derived from botanical, zoological sources, and probiotics demonstrate inhibitory effects on MMP expression, attenuate levels of ROS, and stimulate collagen synthesis, thereby retarding the process of cutaneous aging.

a. *Leontopodium alpinum*, a perennial herb belonging to the family Asteraceae, thrives in harsh weather conditions and is renowned for its bacteriostatic [66], antioxidant [67], blood‐lipid‐regulating [68], anti‐inflammatory [69], and analgesic [70] properties, making it a valuable source of raw material with diverse pharmacological effects. Recent research has demonstrated that the extract derived from *L. alpinum* callus culture effectively mitigates blue‐light‐induced damage in human foreskin fibroblast cells by inhibiting the OPN3‐calcium pathway. Moreover, application of this extract promotes COL1 secretion while reducing levels of ROS and MMP‐1 [71].

b. The ginseng (*Panax ginseng* Meyer) is a medicinal and natural tonic plant belonging to the Araliaceae family. Ginsenosides are considered the primary bioactive constituents of ginseng. While previous studies have primarily focused on the therapeutic effects of ginseng root, recent research has revealed that ginseng leaves contain a higher concentration of ginsenosides, particularly Rg3 and Rk1. These specific compounds have demonstrated their ability to protect human keratinocytes from UVB‐induced damage. Importantly, oral administration of ginseng leaves has been shown to effectively mitigate photoaging in hairless mice by reducing levels of MMP‐2, MMP‐9, IL‐6, and cyclooxygenase‐2 in the skin, thereby diminishing wrinkle formation [72].

c. *Caragana sinica*, a deciduous shrub, has been previously investigated for its potential therapeutic applications in neuralgia, arthritis, and hypertension. It is recognized as a phytoestrogen possessing antibacterial, anti‐inflammatory, neuroprotective, and antioxidant properties [73, 74]. The flower extract of *C. sinica* exerts a positive influence on human keratinocytes (HaCaT cells) by promoting their proliferation and migration through the enhancement of phosphorylation in three conventional MAPK family members (ERK1/2, p38 MAPK, and JNK). These members have been reported to play crucial roles in HaCaT cell proliferation and migration [75, 76, 77, 78, 79, 80, 81]. Moreover, the flower extract also activates the AKT signaling pathway, which is implicated in keratinocyte migration and proliferation [77, 78, 82]. Additionally, it enhances the synthesis of collagen types I and IV while inhibiting the activities of MMP‐2 and MMP‐9. Moreover, there is an upregulation in levels of hyaluronic acid (HA) accompanied by elevated HA synthase‐2 expression [83].

d. The species *Chamaecyparis obtusa* (*C. obtuse*) is extensively cultivated for its high‐quality timber, while the oil derived from *C. obtuse* has been documented to possess antibacterial, anti‐inflammatory, and antioxidant properties [84, 85, 86]. Moreover, an ethanol extract derived from *C. obtusa* exhibited remarkable antioxidant activity and effectively attenuated UVA‐induced fibroblast apoptosis by suppressing the expression levels of MMP‐1, MMP‐3, TNF‐α, and IL‐6 genes. Furthermore, it upregulated mRNA levels of collagen type I and superoxide dismutase [87].

e. The cultivation of *Phaseolus angularis* L. is widely practiced due to the seeds' abundant protein and starch content, as well as their potential in mitigating edema, ameliorating inflammation, and counteracting poisoning [88]. Furthermore, it has been discovered that the extract derived from *P. angularis* L. seeds effectively inhibits the expression of MMP‐1 and MMP‐3, thereby preventing photoaging. This mechanism involves reducing the phosphorylation of p38 and JNK, as well as impeding the nuclear translocation of phosphorylated c‐Fos and c‐Jun upon UVB exposure. Additionally, treatment with *P. angularis* L. seed extract demonstrates reparative properties against UVB‐induced damage while stimulating procollagen type I production [89].

f. The *Cosmos caudatus* Kunth belongs to the Asteracecae family and is cultivated in Malaysia. Its leaves and stems are frequently consumed raw as a salad called *Ulam Raja* or “King's Salad.” In Malaysia, it has become popular due to local beliefs associating regular consumption of *C. caudatus* Kunth with maintaining youthful skin. Extensive research has been conducted on this plant regarding its antioxidant, antidiabetic, anti‐inflammatory, antihypertensive, anti‐obesity, anti‐osteoporotic, and antimicrobial properties [90, 91]. The recent research has demonstrated that extracts derived from *C. caudatus* Kunth exhibit the potential to enhance collagen production by effectively inhibiting collagenase activity. Moreover, it effectively suppresses the expression of MMP‐1 and MMP‐3 while also demonstrating inhibitory effects on NF‐κB activation in CCD‐966SK cells, thereby highlighting its promising role as a potent anti‐aging agent [92].

g. Goji berry (*Lycium barbarum*), a dried fruit of the tree belonging to the Solanaceae family, exhibits anticancer properties [93], enhances immunity [94], improves liver function [95], and inhibits glycation [96]. The fig (*Ficus carica* L.), a member of the Moraceae family, is renowned for its antibacterial, antioxidant, anticancer, antipyretic, emollient, and cholesterol‐lowering properties [97]. *Agastache rugosa* Kuntze, a perennial plant belonging to the *Labiate* family and commonly known as Kwakhyang, Bang‐a or Korean mint, has been found to possess extracts that play a pivotal role in the anti‐photoaging and antioxidative processes of UVB‐irradiated human skin cells and hairless mice [98, 99]. The product known as AGEs Blocker (AB) comprises a blend of goji berry extract, fig extract, and Korean mint extract. Research indicates that AB exerts anti‐aging effects by inhibiting glycation. In hairless mice models treated with AB, there was an observed augmentation in antioxidative enzyme activity accompanied by reduced levels of inflammatory cytokines; downregulation of MMP‐9 expression; elevation in collagen and hyaluronic acid levels resulting in diminished skin wrinkles, improved elasticity, and enhanced hydration [100]. Oral administration of AB in UVB‐irradiated hairless mice effectively attenuated UVB‐induced erythema while simultaneously restoring skin elasticity and reducing wrinkles caused by UVB exposure through upregulation of genes involved in hyaluronic acid synthesis as well as collagen synthesis. Furthermore, AB exhibited inhibitory effects on MMP‐1 and MMP‐9 expressions by suppressing MAPK/AP‐1(c‐fos) activation induced by UVB irradiation [101].

h. Krill oil (KO), derived from *Euphausia superba*, contains unique phospholipids absent in fish oil and constitutes 30%–65% of KO's composition [102]. In addition to its application in human health improvement, krill oil has exhibited potential as a natural marine‐derived ingredient for enhancing skin health. Experimental evidence suggests that krill oil effectively attenuates UVB‐induced skin photoaging by suppressing MMP‐1 production, promoting hyaluronan and collagen synthesis, and inhibiting elastase activity [103].

i. Probiotics, as live microorganisms, have the potential to enhance gut health in humans upon ingestion. Numerous studies have demonstrated their ability to exert systemic effects by modulating the gut environment [104, 105]. Oral administration of *Latilactobacillus sakei* wikim0066 in UVB‐exposed HR‐1 mice led to a reduction in wrinkle formation and epidermal thickness while concurrently promoting procollagen type I synthesis through the regulation of MMP‐1 and MMP‐3, the MAPK signaling pathway, AP‐1 transcription factor, and NF‐κB [106].

j. *Lactobacillus rhamnosus* ATCC 7469 is a probiotic strain that confers numerous benefits for enhancing human health. In studies conducted on mouse skin fibroblast cells, it was observed that this strain exerted protective effects against UVB‐induced DNA damage by downregulating the expressions of MMP‐1, MMP‐2, and MMP‐3 while upregulating the expression of COL1A1. Furthermore, in human epidermal melanocytes, *L. rhamnosus* ATCC 7469 was found to attenuate ROS levels through the Nrf2/Sirt3/SOD2 signaling pathway and inhibit tyrosinase activity via the PKA/CREB/MITF signaling pathway, thereby exhibiting an inhibitory effect on melanin production [107].

In addition to complex natural extracts from plants and animals, there are certain single‐ingredient natural compounds derived from plants that demonstrate inhibitory effects on MMPs.

k. The *Aconitum carmichaelii*, an indigenous plant of East Asia and East Russia, exhibits medicinal properties in terms of fever reduction, asthma alleviation, and sedation [108, 109]. Higenamine, derived from the roots of *Aconitum*, has been employed in the treatment of cardiovascular disorders [110]. Recent investigations have indicated that higenamine, a natural plant alkaloid, may exhibit protective effects against skin aging induced by fine particulate matter in human keratinocytes (HaCaT cells). This compound effectively reduces the production of ROS and mitigates MMP‐1 expression resulting from exposure to fine particulate matter. Moreover, higenamine can interfere with AP1 and NF‐κB activities—two factors closely associated with MMP‐1 transcription [111].

l. The application of hyaluronan as a cosmetic ingredient is extensive, and a novel hyaluronan complex has been developed by combining 30 kDa sodium hyaluronate with its acetylated derivatives. This hyaluronan complex effectively suppressed the expression of MMP‐1 while simultaneously enhancing the expression of type I collagen and dermal–epidermal junction proteins in human dermal fibroblasts [112].

m. The herbaceous plant *Salvia plebeia* R. Br (SP) is abundant in flavonoids and possesses potent anti‐inflammatory and antioxidant properties. Its primary bioactive constituent, homoplantaginin (HP), has been found to exert a protective effect against UVB‐induced damage by attenuating the generation of ROS and malondialdehyde (MDA). Additionally, SP and HP suppress SA‐β‐Gal activity, inhibit the MAPK signaling pathway to reduce MMP‐1 and IL‐6 secretion, as well as activate the TGF‐β/Smad pathway to enhance type I procollagen synthesis [113].

n. The photosensitizer chlorine 6 can be activated by a specific wavelength of light, making it a commonly employed agent in photodynamic therapy (PDT) for targeted cell destruction during tumor treatment [114]. Curcumin, a polyphenolic compound derived from the rhizome of *Curcuma longa* (turmeric), has been extensively investigated for its potential role in UVA and UVB‐induced photoaging [115, 116]. The coadministration of curcumin and chlorine 6 has been documented to augment the efficacy of chlorine 6 photodynamic therapy (PDT) [117]. Recently, three novel derivatives of chlorine 6‐curcumin (Ce6‐propane‐curcumin, Ce6‐hexane‐curcumin, and Ce6‐diPEG‐curcumin) have demonstrated their potential in mitigating photoaging in human skin fibroblasts (Hs68) and mouse embryonic fibroblasts (BALB/c 3T3) cells by modulating the levels of MMP‐1 and MMP‐2. Moreover, these compounds exhibit anti‐inflammatory properties and facilitate collagen synthesis [118].

o. Cycloastragenol (CAG), a triterpene aglycone derived from astragaloside IV extracted from *Astragalus membranaceus* root, exhibits diverse pharmacological effects including enhancement of telomerase activity [119] and involvement in age‐related diseases [120]. The evidence demonstrates that CAG effectively suppresses the expression of MMP‐1, MMP‐9, MMP‐13, and ROS induced by UVB and H_2_O_2_ while simultaneously restoring collagen type I levels. Furthermore, CAG significantly enhances the production of hyaluronic acid as well as crucial hydration factors such as filaggrin and serine palmitoyltransferase (SPT) [121].

p. Hesperidin, a flavanone glycoside, and its aglycone hesperetin are primarily present in bitter oranges (*Citrus aurantium*), peppermint, and select other plants. These compounds have been reported as effective agents in the process of anti‐photoaging [122, 123]. Rhamnose, a monosaccharide found in rutinose, exerts a beneficial role in the process of skin regeneration [124]. When applied to human dermal fibroblasts in combination, hesperidin, hesperetin, rutinose, and rhamnose were observed to attenuate cellular senescence. Hesperidin and hesperetin exhibited inhibitory effects on elastase and hyaluronidase activity, while all four substances tested reduced MMP‐1 and MMP‐2 levels. Moreover, the application of rhamnose and rutinose promoted collagen type I synthesis [125].

### 6.2 The Proteins, Polypeptides, and Amino Acids that Inhibit MMPs

The anti‐aging properties of collagen, whether applied topically or consumed orally, are widely recognized. Furthermore, recent research has revealed the potential of specific proteins, peptides, and amino acids in inhibiting MMP activation and prolonging skin's youthful appearance.

a. The leucine‐rich alpha‐2‐glycoprotein 1 (LRG1) is a member of the leucine‐rich repeat protein family and functions as a secreted glycoprotein. Within the dermal tissue, LRG1 exhibits expression in fibroblasts, keratinocytes, and endothelial cells [126]. Purified recombinant human Leucine‐rich alpha‐2‐glycoprotein 1 (LRG1) has been shown to augment the secretion of collagen type I and suppress the secretion of matrix metalloproteinase‐1 by activating the transforming growth factor‐beta (TGF‐β) signaling pathway. Additionally, a decline in LRG1 mRNA and protein expression has been observed in aged human skin fibroblasts [127]. The SPARC protein is capable of inducing activation of the TGF‐β signaling pathway in human fibroblasts, resulting in enhanced synthesis of collagen type I and reduced secretion of MMP‐1 [128].

b. The photosynthetic protein C‐phycocyanin (C‐PC), derived from *Spirulina*, demonstrates anti‐photoaging properties. Application of topical C‐PC on UVB‐exposed mouse skin resulted in a reduction in sagging and coarse wrinkling, prevention of dermal collagen fiber loosening, enhancement of antioxidant enzyme activity, decrease in expression levels of inflammatory factors, as well as downregulation of MMP‐3 and MMP‐9 [129].

c. The collagen peptide Gly‐Pro‐Val‐Gly‐Pro‐Ser derived from *Oreochromis niloticus* fish exhibited protective effects on photoaging mimic models by enhancing antioxidant enzyme activities and reducing pro‐inflammatory factors both in vitro and in vivo. Furthermore, this peptide upregulated transforming growth factor‐β receptor I, collagen type I, and procollagen type I while downregulating c‐Jun N‐terminal kinase expression. Additionally, it increased hyaluronic acid levels and decreased the protein expressions of ceramide synthase 4 as well as MMP‐1, ‐2, and ‐9. Consequently, it demonstrated efficacy against UVB‐induced skin photoaging [130].

d. γ‐aminobutyric acid (GABA), a non‐protein amino acid endogenously synthesized in the human body [131], plays a crucial role in various skin‐related processes [132, 133]. Substantiated evidence demonstrates the effective inhibition of UVB‐induced MMP‐1 expression by GABA. Moreover, compelling in vivo experiments have demonstrated that supplementation with GABA restores the adverse effects of UVB exposure, including increased skin wrinkles, reduced epidermal thickness, and compromised skin moisture and elasticity [134].

### 6.3 MMP Inhibitors in the Clinical Application

a. The composition of caviar oil includes *Candida Bombicola*/Glucose/Methyl Rapeseedate ferment, *Macadamia ternifolia* seed oil, and caviar extract. It is abundant in docosahexaenoic acid (DHA), eicosapentaenoic acid (EPA), amino acids, vitamins, and minerals [135]. The application of caviar oil demonstrates potential anti‐aging effects through the downregulation of MMP‐1, MMP‐2, and MMP‐9 expression levels while simultaneously promoting elastin and collagen I, III synthesis in human skin tissues. In a clinical study involving a formulation containing 15% caviar oil, significant improvements were observed across various skin parameters including reduction in forehead wrinkles, glabellar lines, nasolabial folds, marionette lines as well as enhancement in water retention capacity, skin hydration levels, elasticity enhancement, clarity improvement, increased dermal density, and overall skin tightening relief [135].

b. Hyaluronic acid (HA) is a renowned and iconic cosmetic ingredient recognized for its anti‐aging properties through the promotion of skin hydration. Numerous studies have demonstrated that the skin penetration ability of HA is contingent upon its molecular weight [136]. In comparison to high molecular weight, intermediate (300 kDa) and low (20–50 kDa) molecular weights of HA demonstrate enhanced dermal penetration [137]. To address the issue of rapid degradation by hyaluronidase in the skin, researchers have developed acetylated HA with enhanced resistance to hyaluronidase degradation and improved deep dermal penetration. Clinical studies have demonstrated the efficacy of acetylated HA in reducing crow's feet and wrinkles in the nasogenian area. Additionally, acetylated HA exhibits inhibitory effects on MMP‐1, MMP‐3, and MMP‐9 expression while attenuating H_2_O_2_‐induced degradation of type I collagen [138].

c. Numerous species of *Rosa* exist, cultivated as horticultural crops for their edible and medicinal petals. A clinical trial demonstrated the efficacy of a formulation containing *Rosa gallica* petals in suppressing MMP‐1 expression, thereby exhibiting anti‐wrinkle and skin whitening properties. In vivo experiments further validated the MMP‐1 inhibiting function of the formulation with *Rosa gallica* petals [139].

d. The rhizomes of *Anemarrhena asphodeloides* Bunge (Liliaceae) have long been utilized in traditional medicine for their antidiabetic, antipyretic, and antidepressant properties, as well as their efficacy in the treatment of febrile diseases [140, 141]. Timosaponin A‐III (TA‐III), identified as the primary chemical constituent of *Anemarrhena asphodeloides* Bunge, has been found to effectively reduce wrinkles in a 12‐week clinical trial involving female subjects aged 43–55 years. Additionally, TA‐III demonstrated inhibitory effects on the upregulation of MMP‐1 expression and pro‐inflammatory cytokines in HaCaT cells exposed to UVB irradiation [142].

e. Autologous serum platelet‐rich plasma (PRP) has been employed for skin rejuvenation in individuals exhibiting wrinkles and signs of aging. A clinical trial conducted on a cohort of healthy female subjects demonstrated the efficacy of PRP treatment in reducing wrinkle appearance, enhancing skin texture, and minimizing pore size. In an experimental setting utilizing a human organotypic skin model, PRP treatment followed by UVB irradiation resulted in diminished expression levels of MMP‐1 and tyrosinase. Moreover, exposure to UVB radiation restored the expression of fibrillin and tropoelastin [143].

f. HDF injections: In the context of aging human skin, the secretion of collagen by dermal fibroblasts diminishes; nevertheless, autologous transdermis injection of human dermal fibroblasts (HDFs) has gained FDA approval as a strategy to impede skin aging. When compared to monolayer culture of HDFs, three‐dimensional spheroids augment the expression of procollagen type I and tissue inhibitor of metalloproteinases‐1 (TIMP‐1), thereby suppressing MMP‐1 expression [144]. The clinical application of HDFs appears to present a safer alternative compared to other small molecular MMP inhibitors.

## 7 Conclusion

Skin aging is a complex process influenced by multiple factors. Extrinsic skin aging can be triggered by environmental elements such as smoking, wind exposure, exposure to harmful chemicals, and an unhealthy diet. Current research predominantly focuses on the role of MMPs in UV‐induced skin damage, with limited attention given to other factors impacting MMPs and skin aging. Recently, investigating the susceptibility of skin to sugar has gained traction as a research endeavor. The formation of AGEs (Advanced Glycation End Products) through protein‐sugar reactions significantly alters the physical, biomechanical, and biological properties of the skin. Accumulation of AGEs in the skin leads to wrinkles, loss of elasticity, dullness, and impaired functionality—one of the primary mechanisms underlying skin aging. Studies have demonstrated that culturing fibroblasts with AGEs upregulates mRNA expression levels of MMP‐8 and MMP‐9 [145]. In a 3D system containing glycated collagen, fibroblasts exhibited reduced quantities but suppressed activity levels of pro‐MMP‐2 [146]. Given the escalating skincare challenges we face, it is imperative to acquire further insights into emerging factors that contribute to disruptions in MMP regulation.

Photoaging is a complex and protracted process involving the continuous degradation, repair, and synthesis of damaged fibers over an extended duration. Therefore, the inhibition of MMPs should be regarded as an ongoing imperative throughout daily life. Table 1 summarizes the natural extracts, protein, and amino acids that could inhibit MMPs as well as the MMP inhibitors in the clinical trials. However, there have been numerous challenges in the clinical application of synthetic small‐molecule inhibitors. As previously mentioned, first‐generation MMP inhibitors exhibit suboptimal safety profiles due to their inadequate pharmacokinetics and lower selectivity. Presently, only periostat (containing doxycycline hydrochloride) has received approval from the U.S. Food and Drug Administration (FDA) as an MMP inhibitor for periodontitis treatment [147]. Most MMP inhibitors exhibit noticeable side effects primarily due to inadequate specificity in inhibitor selection [148, 149]. Moreover, MMPs represent promising therapeutic targets in a wide range of pathological conditions including malignancies, osteoarthritis, neurodegenerative disorders, infectious diseases, and cardiovascular ailments [150, 151, 152, 153, 154]. Moreover, MMPs represent promising therapeutic targets in a wide range of pathological conditions including malignancies, osteoarthritis, neurodegenerative disorders, infectious diseases, and cardiovascular ailments [155, 156, 157, 158]. Consequently, the development of selective MMP inhibitors is imperative rather than employing non‐specific ones.

**TABLE 1:** The inhibitors of MMPs.

Compound	Target	Biological activity	System	References
1. Natural substance extract				
*Leontopodium alpinum* callus culture extract	MMP‐1	Blue‐light‐damage↓ COL‐1 production↑ ROS↓ OPN3 secretion↓	Human foreskin fibroblast cells	Meng, X. et al. 2023
Ginseng leaves	MMP‐2 MMP‐9	Anti‐photoaging↑ Skin wrinkles↓ IL‐6 and cyclooxygenase‐2↓	Human keratinocytes (HaCat) HR‐1 hairless mouse	Son, E. et al. 2023
*Caragana sinica* flower extract	MMP‐2 MMP‐9	HaCat cell proliferation and migration↑ Hyaluronic acid↑ Collagen type I and IV syntheses↑	HaCaT cells	Kim, M.J. et al. 2023
*Chamaecyparis obtusa* (*C. obtuse*) extract	MMP‐1 MMP‐3	Fibroblast death↓ Collagen type I mRNA superoxide dismutase mRNA↑	Human dermal fibroblast cells	Jang, Y.‐A. et al. 2023
*Phaseolus angularis* L. seed extract	MMP‐1 MMP‐3	Anti‐photoaging↑ Antioxidant activities↓ Procollagen type I production↓	HaCaT cells	Oh, S. et al. 2023
*Cosmos caudatus* Kunth extract	MMP‐1 MMP‐3	Anti‐aging↓ Collagenase activity↑ NF‐κB activation↑	CCD‐966SK cells	Loo, Y.C. et al. 2023
AGEs Blocker (Goji berry, Fig, and Korean mint mixed extract)	MMP‐1 MMP‐9	Skin aging↑ Antioxidative enzymes↑ AGEs↓ Collagen and hyaluronic acid↑ Inflammatory cytokines↓	Mouse, Hs68 fibroblasts, HaCaT cells	Yoo, J.H. et al. 2023 Jung, J. et al. 2023
Krill oil	MMP‐1	Oxidative stress↓ Wrinkles↓ Collagen and hyaluronic synthesis↑	DF, HaCaT, and B16/F10 cells Hairless mouse	Kim, J. et al. 2023
*Latilactobacillus sakei* Wikim0066	MMP‐1 MMP‐3	Wrinkle formation↓ Type I procollagen↑ MAPK, AP‐1, and NF‐κB expression↓	Hs68 cells, HR‐1 mice	Park, J.‐Y. et al. 2023
*Lactobacillus rhamnosus* ATCC 7469	MMP‐1 MMP‐2 MMP‐3	DNA damage↓ COL1A1 expression↑ ROS content↓ Tyrosinase activity↓	Mouse skin fibroblast, human epidermal melanocytes	Zhang, X. et al. 2023
Higenamine	MMP‐1	Fine‐dust‐induced skin aging↓ ROS production↓ AP‐1 and NF‐κB activation↓	Human HaCaT cells	Kim, D. et al. 2023
Hyaluronan	MMP‐1	Collagen type I expression↑ Dermal–epidermal junction protein expression↑	Human dermal fibroblasts	Chen, F. et al. 2023
*Salvia plebeia* R. Br (SP) and homoplantaginin (HP)	MMP‐1	ROS, MDA, SA‐β‐Gal, and IL‐6↓ MAPK signaling pathway↓ TGF‐β/Smad pathway↑ Collagen degradation↓	HaCaT cells, mouse	Guo, Y. et al. 2023
Chlorin e6‐Curcumin	MMP‐1 MMP‐2	Photoaging↓ Skin inflammation↓ Collagen synthesis↑	Human skin fibroblasts (Hs68), mouse embryonic fibroblasts (BALB/c 3T3) cells	Thapa Magar, T.B. et al. 2023
Cycloastragenol	MMP‐1 MMP‐9 MMP‐13	ROS generation↓ Collagen type I↑ Hyaluronic acid↑	Human HaCaT cells	Yang, M.H. et al. 2023
Hesperidin, Hesperetin, Rutinose, Rhamnose	MMP‐1 MMP‐2	Elastase, hyaluronidase, and collagenase activity Collagen I production↑	Human dermal fibroblasts	Novotná, R. et al. 2023
2. Protein and amino acids				
Human leucine‐rich alpha‐2‐glycoprotein 1	MMP‐1	Collagen type I secretion↑ TGF‐β signaling pathway↑	Human skin fibroblasts	Park, H.N. et al. 2023
SPARC protein	MMP‐1	Collagen type I secretion↑ TGF‐β signaling pathway↑	Human fibroblasts	Ham, S.M. et al. 2023
C‐phycocyanin	MMP‐3 MMP‐9	Photoaging↓ Collagen fibers loose↑ Antioxidant enzymes activity inflammatory factors↓	Mouse skin	Zhou, Y. et al. 2023
Gly‐Pro‐Val‐Gly‐Pro‐Ser fish collagen peptide	MMP‐1 MMP‐2 MMP‐9	Photoaging↓ Antioxidant enzymes activity↑ Hyaluronic acid↑ c‐Fos, c‐Jun↓ Transforming growth factor‐β receptor I↑ Collagen type I↑	HaCaT cells, mouse	Cho, W. et al. 2023
γ‐aminobutyric acid (GABA)	MMP‐1	Skin wrinkle↓ Epidermal thickness↓ Skin moisture and elasticity↑	In vivo intake by mice	Zhao, H. et al. 2023
3. MMP inhibitors in the clinical trials				
Caviar oil	MMP‐1 MMP‐2 MMP‐9	Aging↓ Elastin production↑ Collagen types I and III↑ Wrinkle↓	Human skin clinical trial Human 3T3‐L1 cells	Le, L.T.T. et al. 2023
Acetylated HA	MMP‐1 MMP‐3 MMP‐9	Anti‐wrinkle↑ Type I collagen degradation	Human skin clinical trial Human skin explants Human dermal fibroblasts	Meunier, M. et al. 2022
*Rosa gallica* petals containing formulation	MMP‐1	Anti‐wrinkle↑ Skin brightness↑ AP‐1 and MAPKs signaling↑ Pathways ↓	Clinical trial in human skin Human skin tissue samples	Song, Y.‐R. et al. 2020
Timosaponin A‐III	MMP‐1	Wrinkle↓ Photoaging↓ Pro‐inflammatory cytokines expression↓ TIMP expression↑	Human skin clinical trial HaCat cells	Im, A.R., et al. 2020
Autologous serum platelet‐rich plasma (PRP)	MMP‐1	Wrinkles, texture and pores↓ Tyrosinase↓ Fibrillin expression↑ Tropoelastin expression↑	Human skin clinical trial Human organotypic skin model	Du, R. and Lei, T. 2020
Autologous transdermis injection of human dermal fibroblasts	MMP‐1	Procollagen type I↑ TIMP expression↑	Human skin clinical trial	Hu, S., et al. 2019

Due to the necessity for enhanced security measures, there is a growing inclination toward investigating natural sources of MMP inhibitor substances. The extraction of MMP inhibitors from plants and animals offers the potential to obtain these inhibitors through dietary supplements rather than pharmaceutical interventions, thereby presenting a safer approach to inhibiting MMPs in skin aging and maintaining youthful skin. As previously mentioned, numerous natural substances have demonstrated their ability to inhibit MMPs. Although certain plant extracts have been observed to reduce the expression of MMPs, the specific active ingredients responsible for this effect remain unidentified. It is plausible that by harnessing the known active ingredients found in food, drugs, and cosmetics, it may be possible to enhance the inhibitory effects on MMPs. Additionally, studying isolated individual active ingredients can significantly contribute toward developing targeted MMP inhibitors.

## Author Contributions

Conceptualization: Chunyue Zhao, Xianglong Chen, Xiuqing Yin, and Yanfei Jiang; Investigation: Chao Feng, Xianglong Chen, and Xiuqing Yin; Writing – Original Draft: Chao Feng and Chunyue Zhao; Writing – Review & Editing: Chao Feng and Chunyue Zhao; Supervision: Yanfei Jiang.

## Ethics Statement

It is not applicable because this study is based exclusively on published literature.

## Conflicts of Interest

The authors declare no conflicts of interest.

## Data Availability

Data sharing not applicable to this article as no datasets were generated or analysed during the current study.
